# Telemedicine Adoption Among Health Care Professionals in Israeli Geriatric Medical Centers: Moderated Mediation Cross-Sectional Questionnaire Study

**DOI:** 10.2196/95371

**Published:** 2026-10-05

**Authors:** Gizell Green, Alena Lochmannová, Riki Tesler, Tammy Porat-Packer

**Affiliations:** 1 Nursing Department, The Max Stern Yezreel Valley College, D.N. Emek Yezreel, Emek Yezreel, Northern District, 1930600, Israel, 972 50-9398808; 2 Shoham Medical Center, Pardes Hana Karkur, Haifa District, Israel; 3 Department of Crisis Management, Faculty of Security Management, Police Academy of the Czech Republic in Prague, Prague, Czech Republic; 4 Department of Health Systems Management, Faculty of Health Sciences, Ariel University, Ariel, Judea and Samaria Area, Israel

**Keywords:** telemedicine, anxiety, health personnel, geriatrics, surveys and questionnaires, technology acceptance, Israel

## Abstract

**Background:**

Telemedicine adoption among health care professionals is hindered by resistance to change (RTC), comprising routine-seeking, emotional reactivity, short-term focus, and cognitive rigidity, which interacts with perceived ease of use (PEU) and anxiety within the Technology Acceptance Model framework.

**Objective:**

This study aimed to test a moderated mediation model in which PEU mediates the association between RTC, assessed both overall and by dimension, and intention to use telemedicine, while telemedicine-related anxiety moderates the association between PEU and intention to use. Participants were recruited from 4 governmental geriatric medical centers in Israel.

**Methods:**

A cross-sectional study was conducted among 377 health care professionals from 4 Israeli governmental geriatric medical centers. Telemedicine use in the participating centers was limited, optional, and consisted mainly of remote patient monitoring. Data collection was conducted between March and June 2024. Data were collected using validated self-administered questionnaires measuring telemedicine-related anxiety, RTC with 4 dimensions (routine seeking, emotional reactivity, short-term focus, and cognitive rigidity), PEU, and intention to use telemedicine. Statistical analyses were performed using SPSS (version 28) and PROCESS Model 14 for moderated mediation analysis.

**Results:**

The moderated mediation analysis revealed that RTC was negatively associated with the intention to use telemedicine through the lower PEU, with this indirect pathway being attenuated but not eliminated by high telemedicine-related anxiety. The overall indirect effect remained negative and statistically significant across all anxiety levels.

**Conclusions:**

Health care professionals’ RTC reduced their intention to use telemedicine by lowering PEU, with telemedicine-related anxiety moderating but never eliminating this relationship. These findings suggest that telemedicine implementation in geriatric settings should consider both RTC and PEU. The positive indirect association observed for cognitive rigidity was unexpected and should be replicated before specific practical recommendations are made.

## Introduction

### Background

Telemedicine, defined as the use of telecommunications technology to provide medical care and health services remotely [1], has emerged as a transformative force in health care delivery [2,3]. This technology encompasses a variety of applications, including virtual consultations, remote patient monitoring, and mobile health apps, which allow health care providers to diagnose, treat, and manage patients without the need for an in-person visit [4]. Despite its potential to increase access to health care, improve patient outcomes, and enhance the efficiency of health care delivery as documented by Alotaibi and Federico [5], the World Health Organization [6], and Bashshur et al [7], telemedicine has faced inconsistent uptake among health care professionals due to various barriers [8,9], with resistance to change (RTC) emerging as a particularly significant obstacle within the Technology Acceptance Model (TAM) framework [10].

RTC represents a multifaceted construct encompassing routine seeking, emotional reactivity, short-term focus, and cognitive rigidity [11-13]. More specifically, it refers to the emotional and behavioral response of individuals or groups when faced with new ideas, practices, or technologies [14-16]. This resistance can manifest as skepticism, reluctance, or outright opposition to adopting changes, often hindering the implementation of new initiatives or technologies, such as telemedicine in health care settings [17]. Oreg [13,18] developed the RTC scale, which was later validated across 17 nations [19], demonstrating its universal applicability. Understanding the dimensions of routine seeking, emotional reactivity, short-term focus, and cognitive rigidity of this construct is crucial for addressing barriers to telemedicine adoption.

The first dimension, routine seeking, reflects the tendency of individuals to prefer established habits and processes over new methods or technologies [20]. This preference often arises from a desire for stability and familiarity, leading to reluctance in embracing changes that disrupt existing routines, as noted by Kotter [21]. The second dimension, emotional reactivity, plays a crucial role as it encompasses the immediate emotional responses individuals experience when faced with change [14,15]. Emotions such as fear, anxiety, or frustration can significantly impact one’s willingness to adopt new practices or technologies [22]. The third dimension, short-term focus, is characterized by the inclination to prioritize immediate outcomes and benefits over long-term gains. Individuals with a short-term perspective may resist change due to their concern for the immediate implications of new practices, often overlooking potential future advantages [17]. Finally, cognitive rigidity represents a key element, reflecting the inability or unwillingness to adapt one’s thinking or behavior in response to new information or changing circumstances. This rigidity can result in a steadfast adherence to old ways of thinking, making it challenging for individuals to accept and implement change [13].

The adoption and use of telemedicine technologies have been significantly influenced by the interaction between RTC, perceived ease of use (PEU), and anxiety [17,23]. Within the TAM framework, research has shown that the PEU of technology significantly influences an individual’s intention to use it, particularly in health care settings [17,24]. The TAM, originally developed by Davis [10], posits that PEU and perceived usefulness are primary determinants of technology acceptance. Recent extensions of TAM and related dual-factor models have added inhibiting factors, including RTC and technology-related anxiety, to the original focus on facilitating beliefs such as PEU [14,17,24]. When users perceive a technology as user-friendly, their likelihood of adopting it increases, as they feel more confident in navigating its features [25]. However, this relationship can be moderated by anxiety related to using technology. Individuals who experience higher levels of anxiety may struggle to engage with even the simplest digital platforms, leading to a decrease in their intention to use such technologies [26,27]. This relationship suggests that while ease of use is a critical factor in technology adoption, addressing the psychological barriers, such as anxiety, is essential for enhancing user intent [28].

Anxiety can significantly impede an individual’s perception of ease of use when engaging with telemedicine technologies, as heightened feelings of apprehension may lead to a lack of confidence in navigating these platforms [26]. When health care professionals experience heightened anxiety about adopting new technologies, their perception of these technologies’ ease of use tends to decline [29]. Consequently, this diminished perception of ease can adversely affect the intention to use telemedicine services, as users may avoid adopting technologies that they find intimidating or challenging to operate [30]. Furthermore, prior studies have reported an association between RTC and intention to use telemedicine [20,31] as well as an indirect association through PEU [32].

Research has also associated telemedicine-related anxiety with less favorable usability perceptions and weaker intentions to use digital technologies [26,27,29,30,33]. However, previous studies have generally examined these relationships separately, focusing on RTC and telemedicine acceptance [17,24], PEU and intention to use [23], or telemedicine-related anxiety and technology acceptance [27,33]. It is therefore unclear whether telemedicine-related anxiety changes the strength of the association between PEU and intention to use and whether the indirect association between RTC and intention to use through PEU differs across anxiety levels.

Previous studies have also tended to treat RTC as a single overall construct rather than examining its 4 dimensions separately. Although RTC comprises routine seeking, emotional reactivity, short-term focus, and cognitive rigidity [13,18,19], limited evidence is available on whether these dimensions show the same pattern in relation to telemedicine acceptance. Reliance on an overall RTC score may conceal dimension-specific associations with PEU and intention to use. Governmental GMCs provide a theoretically relevant setting for this model because telemedicine must be incorporated into established multidisciplinary workflows rather than adopted as a stand-alone technology [11,34]. In this setting, resistance may shape perceptions of ease of use, while anxiety may weaken the association between PEU and intention to use. The present study extends recent TAM and dual-factor models by examining RTC both as an overall construct and as 4 separate dimensions within 1 conditional process model. It tests whether RTC is associated with intention to use through PEU, whether this indirect association differs across anxiety levels, and whether the same pattern applies to all 4 RTC dimensions [32,35,36].

### Main Research Question and Hypotheses

The main research question is as follows: Is the indirect relationship between RTC (overall and its subcategories: routine seeking, emotional reactivity, short-term focus, and cognitive rigidity) and intention to use telemedicine via the mediator ease of use moderated by anxiety among health care professionals in governmental geriatric hospitals in Israel?

Accordingly, the hypotheses are as follows:

- H1: Higher RTC, as well as higher levels of each dimension, will be associated with lower PEU of telemedicine.
- H2: Higher PEU will be associated with a stronger intention to use telemedicine.
- H3: PEU will mediate the association between RTC and intention to use telemedicine. Specifically, higher RTC will be associated with lower PEU, which, in turn, will be associated with a weaker intention to use telemedicine. This indirect association will also be examined for 4 dimensions of RTC.
- H4: Telemedicine-related anxiety will moderate the indirect association between RTC and intention to use telemedicine through PEU. Specifically, the indirect association will become weaker as telemedicine-related anxiety increases.

## Methods

This research used a cross-sectional study design to examine the phenomenon at a single point in time [37]. The study methodology adhered to the STROBE (Strengthening the Reporting of Observational Studies in Epidemiology) guidelines to ensure methodological rigor and reporting transparency [38]. A completed STROBE checklist is provided in Checklist 1.

### Participants

Study participants comprised health care professionals employed in four Israeli governmental GMCs: Dorot Medical Center, Fliman Medical Center, Shmuel Harofeh Medical Center, and Shoham Medical Center. The sample consisted of 377 health care professionals representing caregivers, including physicians, nurses, physiotherapists, speech-language therapists, occupational therapists, and clinical dietitians. At the time of data collection, the target population in these 4 centers totaled 1184 professionals: 146 physicians, 790 nurses, and 248 allied health professionals.

Participant inclusion criteria encompassed health care professionals (physicians, nurses, and multidisciplinary staff including physiotherapists, speech therapists, occupational therapists, and clinical dietitians) employed within the specified governmental GMCs (Dorot, Fliman, Shmuel Harofeh, and Shoham), maintaining employment tenure of at least 6 months, demonstrating Hebrew language proficiency, and providing voluntary informed consent for participation. At the time of data collection, telemedicine use in the participating centers was limited and optional and consisted mainly of remote patient monitoring. The questionnaire assessed telemedicine in general and did not distinguish among audio-only, video-based, remote-monitoring, or hybrid modalities, or between dyadic and triadic interactions. Telemedicine use was neither mandated nor actively discouraged by hospital management. Exclusion criteria consisted of health care professionals with less than 6 months of employment at the GMCs, insufficient Hebrew language competency, and declined consent for research participation. The study sought to reach the entire staff population. Visits were conducted in every department across all work shifts, and questionnaires were offered to all staff present. A total of 377 professionals completed the questionnaire, corresponding to 31.8% of the 1184 professionals employed in the 4 centers. The 377 questionnaires included in the analyses were fully completed. The sample’s professional composition was similar to that of the target population (nurses: n=256, 67.9% vs physicians: n=46, 12.2% vs allied health: n=75, 19.9%).

### Procedure

Data collection operations were supervised by a designated research coordinator responsible for managing the comprehensive data gathering process across all participating GMCs. The research coordinator maintained ongoing communication with field surveyors and medical center administration while providing oversight for the complete quantitative data collection protocol.

The data acquisition was conducted through self-administered questionnaires distributed by trained student surveyors from the University’s School of Health Sciences. Survey implementation followed prescheduled department visits coordinated with departmental supervisors, during which staff members were invited to complete anonymous self-report questionnaires addressing the research variables. Supervisors and hospital managers were not present during questionnaire completion, and data were collected by independent academic surveyors who were not employed by the participating centers. Questionnaires were completed privately in designated rooms and were collected anonymously. Data collection was conducted between March and June 2024 across the 4 participating governmental GMCs.

Upon questionnaire completion, surveyors secured all materials in sealed envelopes and transferred them to the research coordinator for subsequent coding and data entry procedures. This systematic approach ensured data integrity and maintained participant anonymity throughout the collection process.

### Instruments

The data collection for this study was carried out using a structured questionnaire that included 5 essential measurement scales designed to assess various aspects of telemedicine use and the participants’ reactions.

The first measurement scale focused on telemedicine-related anxiety. Anxiety levels concerning the use of telemedicine in patient care settings were evaluated through a 4-item instrument based on the work of Venkatesh et al [27]. A sample item in this scale was as follows: “I experience concern when using telemedicine in patient care contexts.” Participants’ responses were captured on a 6-point scale, with 1 indicating strong disagreement and 6 indicating strong agreement. The reliability of this scale, measured by Cronbach α, was found to be *α*=.89, suggesting a high level of internal consistency.

The second measurement scale addressed RTC, a critical factor in understanding how health care professionals adapt to new technologies such as telemedicine. This scale was based on Oreg [18] 17-item instrument, which examines 4 distinct dimensions of change resistance.

The first dimension, routine seeking, was measured using 5 items. A sample item from this dimension was as follows: “I typically view changes (such as telemedicine implementation) negatively within my workplace.” The reliability of this scale was *α*=.75, indicating moderate internal consistency.

The second dimension, emotional reactivity, consisted of 4 items. A sample item in this category was as follows: “Learning about significant workplace changes (like telemedicine adoption) would likely increase my stress levels.” This dimension had a reliability of *α*=.84, demonstrating strong internal consistency.

The third dimension, short-term focus, was assessed using 4 items. An example item was as follows: “Workplace plan modifications (including telemedicine integration) create considerable inconvenience for me.” This dimension demonstrated a high level of reliability with *α*=.88.

The final dimension, cognitive rigidity, was evaluated using 3 items. A sample item for this dimension was as follows: “I rarely modify my opinions once formed.” Initially, the reliability for this dimension was suboptimal, with *α*=.58. However, after removing one poorly performing item, the reliability increased to an acceptable level, with *α*=.75.

Each of these scales was carefully selected to provide comprehensive insight into the factors influencing health care professionals’ attitudes toward telemedicine and their readiness for its integration into their work environments.

The full scale for measuring RTC showed satisfactory internal consistency (*α*=.86). The scale used a 6-point response format (1=strongly disagree, 6=strongly agree) for all items.

Perceptions of telemedicine’s ease of use were assessed using a 6-item adaptation of Davis [10] PEU scale. On a 7-point Likert continuum (1=“strongly disagree,” 7=“strongly agree”), respondents evaluated statements such as “Acquiring the skills needed to use telemedicine in my workplace would be straightforward for me.” The scale demonstrated excellent internal consistency (*α*=.94). Furthermore, intentions to adopt telemedicine were measured with a 3-item behavioral-intention instrument, also derived from Davis [10]. Using the same 7-point response format, participants indicated their agreement with items like “I plan to incorporate telemedicine into my professional practice within the next year.” Reliability for this measure was outstanding (*α*=.95).

To place these perceptions and intentions in context, a structured demographic questionnaire captured key personal and professional characteristics, including age, gender, highest educational qualification, professional role, years of health care experience, and current employment status within geriatric care.

All instruments were translated from English into Hebrew and back-translated into English, with discrepancies reviewed and resolved to ensure semantic and conceptual equivalence. The instruments were based on previously validated scales; prior to the main data collection, the Hebrew versions were pilot-tested among health care professionals working in governmental geriatric facilities to assess item clarity and cultural appropriateness, while internal consistency was evaluated in the full study sample.

### Statistical Analysis

Statistical analyses were performed using the SPSS software (version 28).

Prior to analysis, all questionnaires were screened for missing values at both the item and scale levels, and no missing values were identified on any study variable. This completeness is attributable to the mode of administration: questionnaires were completed in the presence of a trained surveyor, who remained available to clarify any item that respondents found unclear and reviewed each questionnaire for unanswered items upon collection. Accordingly, all 377 cases contributed to every analysis.

Multicollinearity was assessed separately for each moderated mediation model using variance inflation factor (VIF) and tolerance statistics. VIF values below 5 and tolerance values above 0.20 were considered acceptable.

Analytical procedures encompassed descriptive statistical measures, frequency distributions, percentage calculations, internal consistency assessments, and correlation analyses. The research hypotheses were evaluated through PROCESS Model 14 (version 4.3) to examine moderated mediation pathways. Separate moderated mediation analyses were also conducted for each RTC dimension (routine seeking, emotional reactivity, short-term focus, and cognitive rigidity) to examine whether the moderated mediation pattern was consistent across the 4 dimensions. These analytical approaches enabled a comprehensive investigation of the relationships among study variables while accounting for the proposed moderated mediation effects within the theoretical model.

### Ethical Considerations

Ethical approval for this investigation was obtained from the Ethics Committee of the School of Health Sciences, Ariel University, under protocol code AU-HEA-GG-20231110‐1. Study participants received comprehensive information regarding research objectives and were provided with assurances concerning data confidentiality and participant anonymity. Participation remained entirely voluntary, with individuals retaining the right to discontinue involvement at any point without penalty or adverse consequences. All participants provided written informed consent before participation.

Data security protocols were implemented to ensure secure storage, with access restricted exclusively to authorized research personnel. Publication of findings adhered to aggregate reporting standards, eliminating any identifying information pertaining to individual participants or participating health care institutions. These measures ensured full compliance with research ethics standards and participant protection requirements.

## Results

The mean age varied across professional groups: physicians (mean 40.66, SD 11.51 y), nurses (mean 42.73, SD 12.86 y), and other multidisciplinary staff (mean 37.25, SD 10.02 y), with nurses being the oldest group on average and multidisciplinary staff being the youngest. For other background characteristics, see Table 1.

**Table 1.** Background characteristics by professional groups^a^.

Characteristic and categories	Nurses (n=256, 67.9%), n (%)	Physicians (n=46, 12.2**%**), n (%)	Multidisciplinary staff (n=75, 19.9%), n (%)	Chi-square (*df*)
Children				11.09^b^ (2)
Yes	192 (75.0)	24 (52.2)	48 (64.0)	
No	64 (22.3)	22 (42.9)	27 (36.0)	
Religion				45.39^c^ (8)
Jewish	108 (42.2)	15 (32.6)	55 (73.3)	
Muslim	117 (45.7)	23 (50)	16 (21.3)	
Christian	17 (6.6)	1 (2.2)	2 (2.7)	
Druze	0 (0)	0 (0)	1 (1.3)	
Other	14 (5.5)	7 (15.2)	1 (1.3)	
Level of religiosity				38.85^c^ (8)
Secular	75 (29.3)	21 (45.7)	42 (56.0)	
Traditional	110 (43.0)	12 (26.1)	11 (14.7)	
Religious	50 (19.5)	6 (13.0)	18 (24.0)	
Very religious	7 (2.7)	0 (0)	2 (2.7)	
Other	14 (5.5)	7 (15.2)	2 (2.7)	
Work shifts				155.56^c^ (2)
Morning	51 (19.9)	28 (59.5)	69 (93.2)	
Evening or night shifts	205 (80.1)	18 (40.5)	6 (6.8)	

a Percentages are calculated within professional groups.

b *P*<.01.

c *P*<.001.

Table 1 shows that most nurses (n=192, 77.7%) and multidisciplinary staff (n=48, 64.0%) had children, compared to 57.1% (n=24) of the physicians. Religious affiliation varied significantly, with multidisciplinary staff being predominantly Jewish (n=55, 73.3%), while nurses and physicians showed more diversity between Jewish and Muslim affiliations. The majority of nurses worked evening or night shifts (n=205, 84.4%), whereas most physicians (n=28, 59.5%) and particularly multidisciplinary staff (n=69, 93.2%) worked morning shifts.

Multicollinearity diagnostics indicated no problematic multicollinearity across the 5 models, with VIF values ranging from 1.064 to 1.504 and tolerance values ranging from 0.665 to 0.940.

Table 2 shows that telemedicine-related anxiety demonstrated positive correlations with RTC variables, particularly with emotional reactivity (*r*=0.55; *P*<.001) and routine seeking (*r*=0.51; *P*<.001). Conversely, PEU showed negative correlations with all resistance dimensions except cognitive rigidity, which exhibited a positive correlation (*r*=0.28; *P*<.001). The strongest relationship emerged between PEU and intention to use telemedicine (*r*=0.69; *P*<.001).

Before evaluating the research hypotheses through PROCESS Model 14 (version 4.3) to examine moderated mediation pathways, all variables were standardized (*z* scores) to facilitate interpretation. The index of moderated mediation for the indirect association between RTC and intention to use through ease of use was statistically significant (index=0.016, 95% CI 0.003-0.035), supporting the presence of a moderated indirect effect. Table 3 presents a detailed description of all analysis steps.

**Table 2.** Correlation matrix, means, and SDs for model variables.

Variable	1	2	3	4	5	6	7	8
Routine seeking	1.00	0.68^a^	0.70^a^	0.13^b^	0.86^a^	0.51^a^	−0.31^a^	−0.32^a^
Emotional reactivity	0.68^a^	1.00	0.74^a^	0.16^a^	0.87^a^	0.55^a^	−0.27^a^	−0.23^a^
Short-term focus	0.70^a^	0.74^a^	1.00	0.16^a^	0.88^a^	0.43^a^	−0.30^a^	−0.25^a^
Cognitive rigidity	0.13^b^	0.16^a^	0.16^a^	1.00	0.40^a^	0.10	0.28^a^	0.12^b^
Total RTC^c^	0.86^a^	0.87^a^	0.88^a^	0.40^a^	1.00	0.54^a^	−0.24^a^	−0.25^a^
Telemedicine-related anxiety	0.51^a^	0.55^a^	0.43^a^	0.10	0.54^a^	1.00	−0.31^a^	−0.31^a^
PEU^d^	−0.31^a^	−0.27^a^	−0.30^a^	0.28^a^	−0.24^a^	−0.31^a^	1.00	0.69^a^
Intention to use telemedicine	−0.32^a^	−0.23^a^	−0.25^a^	0.12^b^	−0.25^a^	−0.31^a^	0.69^a^	1.00
Mean (SD)	3.03 (1.00)	3.01 (1.11)	2.61 (1.16)	3.91 (1.15)	3.08 (0.85)	3.50 (1.39)	4.53 (1.25)	4.01 (1.46)

a *P*<.001.

b *P*<.05.

c RTC: resistance to change.

d PEU: perceived ease of use.

**Table 3.** Moderated mediation model predicting intention to use through ease of use, with anxiety as moderator.

Model components	*B* (SE; 95% CI)	*t* test (*df*)	*P* value
Predicting the mediator: ease of use			
RTC^a^	−0.24 (0.05; −0.34 to −0.14)	−4.72 (375)	<.001
Conditional effects of ease of use on intention at different anxiety levels			
Low	0.7 (0.05; 0.61 to 0.80)	14.4 (372)	<.001
Mean	0.62 (0.04; 0.54 to 0.70)	15.01 (372)	<.001
High	0.56 (0.06; 0.45 to 0.68)	9.58 (372)	<.001
Predicting the dependent variable: intention to use			
Resistance	−0.05 (0.04; −0.14 to 0.04)	−1.05 (372)	.29
Ease of use	0.63 (0.04; 0.55 to 0.71)	15.88 (372)	<.001
Conditional indirect effects at different anxiety levels			
Low	−0.17 (0.05; −0.27 to −0.08)	—^b^	—
Mean	−0.15 (0.04; −0.24 to −0.07)	—	—
High	−0.14 (0.04; −0.22 to −0.06)	—	—

a RTC: resistance to change.

b Not applicable.

RTC was negatively associated with ease of use (*B*=−0.24, SE=0.05, *t*_375_=−4.72; *P*<.001), which in turn was positively associated with intention to use (*B*=0.63, SE =0.04, *t*_372_=15.88; *P*<.001). The significance of the mediation effect was estimated using 95% CIs calculated based on 5000 bootstrap samples. Since zero was not included in the CIs at all levels of anxiety, the indirect relationships were significant: anxiety (indirect effect =−0.15, 95% CI −0.236 to −0.072).

The interaction between ease of use and anxiety was significant (*B*=−0.07, SE=0.03, *t*_372_=−1.98; *P=*.04). The decomposition of the interaction into conditional effects of ease of use on intention at different levels of anxiety demonstrates that the positive relationship between ease of use and intention to use weakened as anxiety increased: low anxiety (*B*=0.70, SE=0.05, *t*_372_=14.40; *P*<.001), mean anxiety (*B*=0.62, SE=0.04, *t*_372_=15.01; *P*<.001), and high anxiety (*B*=0.56, SE=0.06, *t*_372_=9.58; *P*<.001).

The conditional indirect relationships show that the negative effect of RTC on intention to use through reduced ease of use weakens as anxiety increases. However, even at high anxiety levels, the indirect relationship remains significant. Therefore, it appears that while anxiety moderates the strength of the indirect effect, it cannot eliminate it entirely.

Figure 1 illustrates how anxiety moderates the relationship between ease of use and intention to use. Three lines represent different levels of anxiety: low (1 SD below the mean), mean, and high (1 SD above the mean). The positive relationship between ease of use and intention to use is the strongest at low anxiety levels (slope =0.70) and weakest at high anxiety levels (slope =0.56), though the relationship remains positive and significant across all anxiety levels. This pattern suggests that while ease of use was consistently associated with intention to use, the magnitude of this association was smaller at higher levels of anxiety.

**Figure 1.**
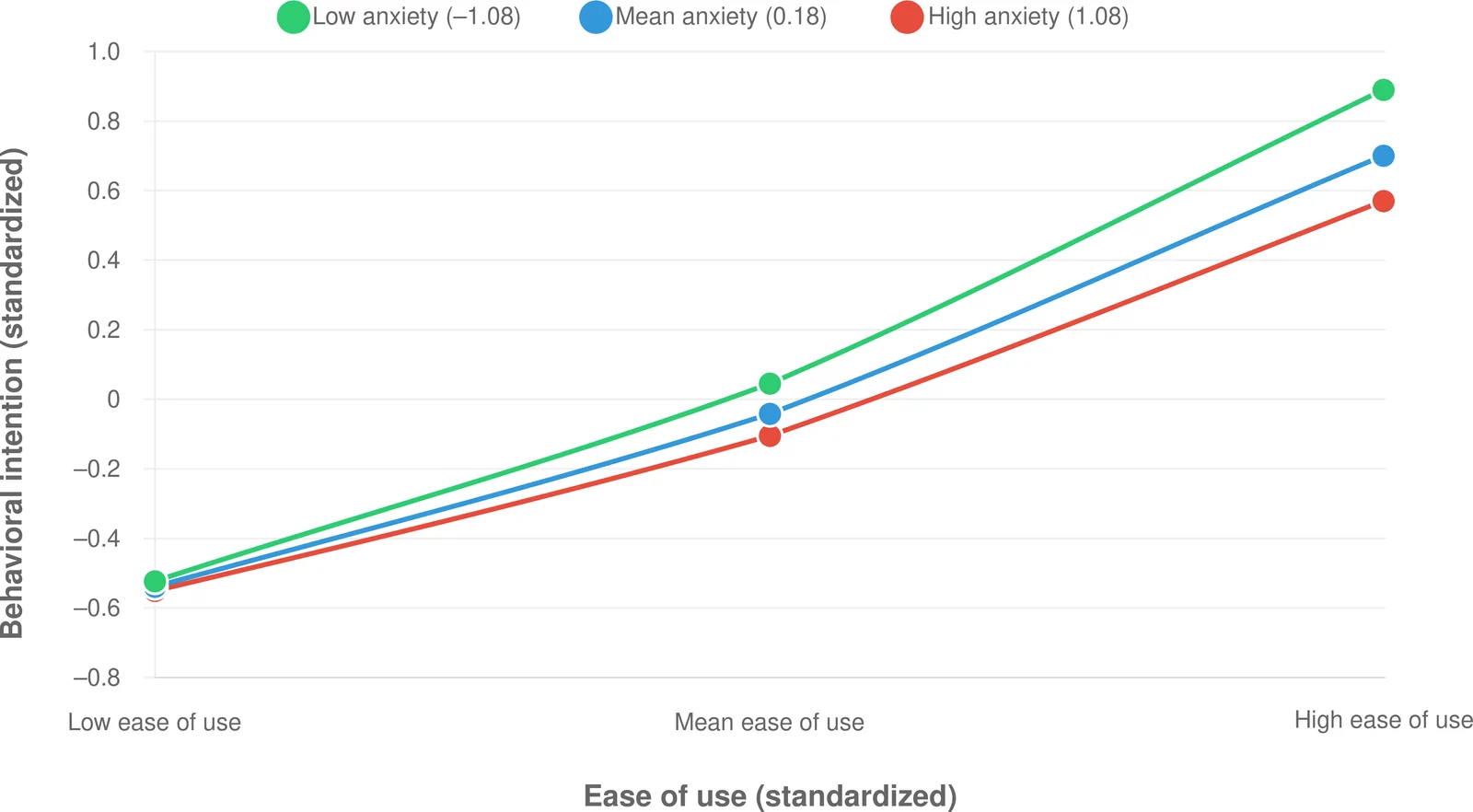
The moderating effect of anxiety on the relationship between ease of use and intention to use.

Figure 2 shows that the negative indirect association between RTC and intention to use through ease of use decreases as anxiety increases, from −0.17 at low anxiety to −0.14 at high anxiety. All indirect effects are statistically significant, as indicated by CIs that do not include zero. The pattern demonstrates that while anxiety reduces the indirect effect, it remains significant across all anxiety levels examined.

The hypothesized moderated mediation model, in which ease of use mediated the association between RTC and intention to use, with anxiety moderating the association between ease of use and intention to use, is presented in Figure 3.

**Figure 2.**
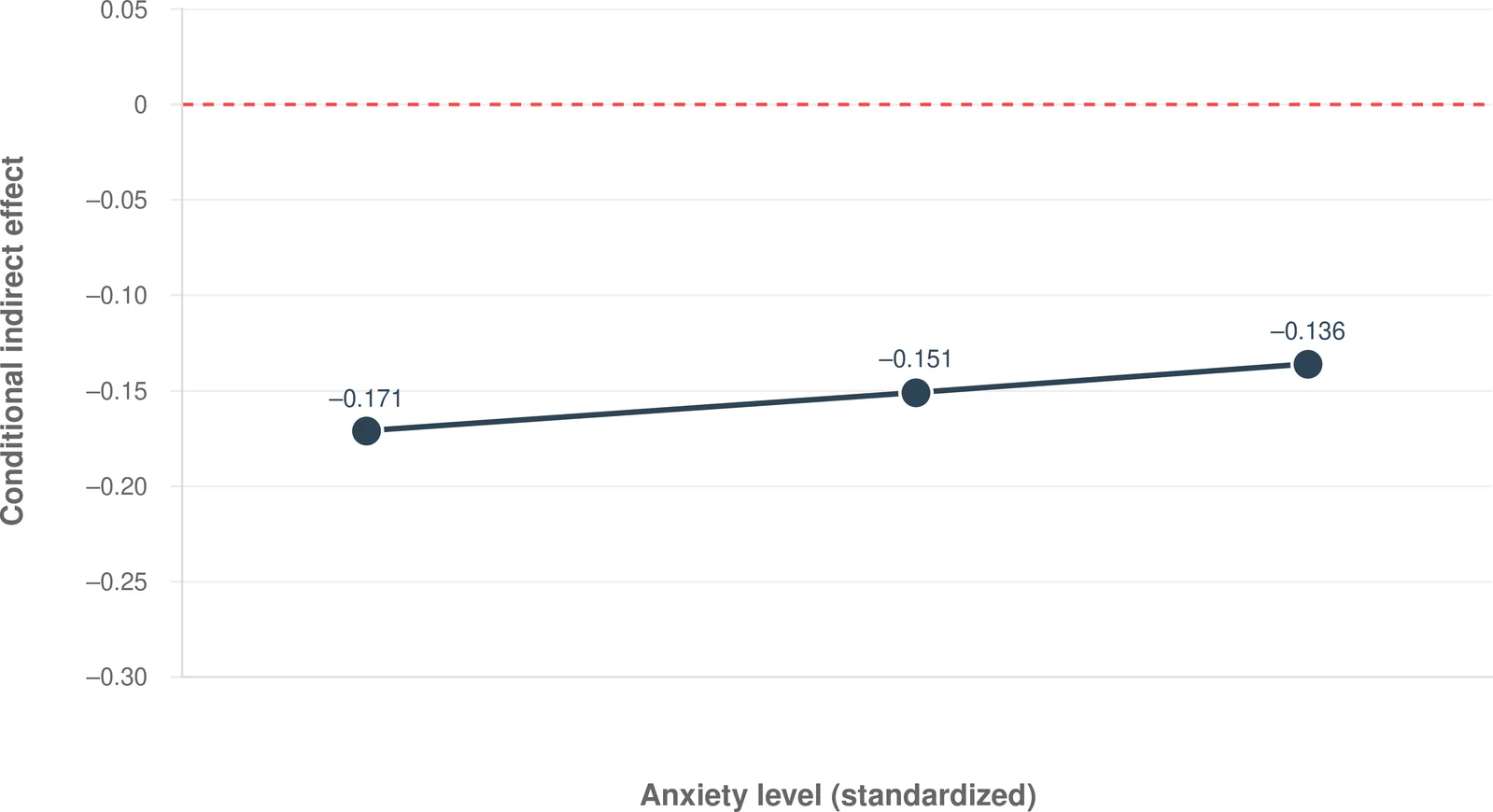
Conditional indirect effects of resistance to change (RTC) on intention to use through ease of use at different levels of anxiety.

Following the main analysis, each of the 4 dimensions of RTC (routine seeking, emotional reactivity, short-term focus, and cognitive rigidity) was examined separately as an independent variable using Model 14 to test the moderated mediation hypothesis.

The index of moderated mediation for the indirect relationship between routine seeking and intention to use through ease of use, with anxiety as the moderator, was statistically significant (index =0.020, 95% CI 0.003-0.042), supporting the presence of a moderated indirect effect. Table S1 presents a detailed description of all analysis steps in Multimedia Appendix 1.

Routine seeking was negatively associated with ease of use (*B*=–0.32, SE=0.05, *t*_375_=−6.41; *P*<.001), which in turn was positively associated with intention to use (*B*=0.62, SE=0.04, *t*_372_=15.50; *P*<.001). The significance of the mediation effect was estimated using 95% CIs calculated based on 5000 bootstrap samples. Since zero was not included in the CIs at all levels of anxiety, the indirect relationships were significant across all anxiety levels.

**Figure 3.**
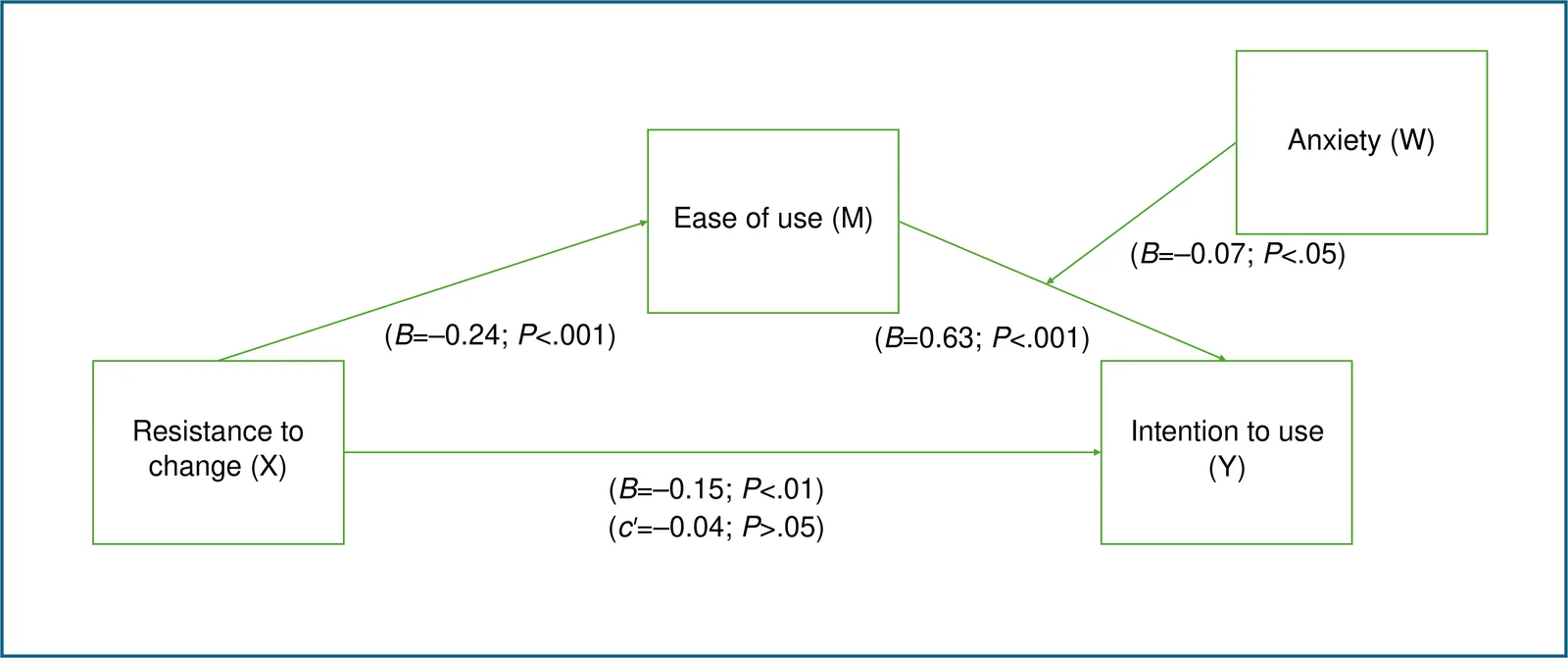
The research model.

As presented in the results, the interaction between ease of use and anxiety was not significant (*B*=−0.06, SE=0.03, *t*_372_=−1.83; *P*=.07). Although the simple interaction effect was marginally nonsignificant, Hayes [39] argues that the index of moderated mediation is the more appropriate and sensitive test for detecting moderation in the indirect pathway, as it directly examines moderation of the mediated effect through bootstrap procedures rather than relying on the traditional interaction test which may lack sufficient power to detect subtle moderation effects.

The conditional effects show that ease of use remains positively associated with intention to use across all anxiety levels: low anxiety (*B*=0.69, SE=0.05, *t*_372_=13.84; *P*<.001), mean anxiety (*B*=0.61, SE=0.04, *t*_372_=14.71; *P*<.001), and high anxiety (*B*=0.56, SE=0.06, *t*_372_=9.53; *P*<.001). The conditional indirect relationships show that the negative effect of routine seeking on intention to use through reduced ease of use weakens as anxiety increases: low anxiety (indirect effect =−0.22, 95% CI −0.30 to −0.15), mean anxiety (indirect effect=−0.20, 95% CI −0.27 to −0.13), and high anxiety (indirect effect=−0.18, 95% CI −0.26 to −0.11). All indirect effects remained statistically significant across anxiety levels, indicating that while anxiety moderates the strength of the indirect effect, it cannot eliminate it entirely.

The index of moderated mediation for the indirect relationship between emotional reactivity and intention to use through ease of use, with anxiety as the moderator, was statistically significant (index=0.019, 95% CI 0.003-0.040), supporting the presence of a moderated indirect effect. Table 4 presents a detailed description of all analysis steps.

**Table 4.** Moderated mediation model predicting intention to use through ease of use, with anxiety as moderator.

Model component	*B* (SE; 95% CI)	*t* test (*df*)	*P* value
Predicting the mediator: ease of use			
Emotional reactivity	−0.27 (0.05; −0.38 to −0.17)	−5.38 (375)	<.001
Predicting the dependent variable: intention to use			
Emotional reactivity	0.003 (0.05; −0.09 to 0.09)	.06 (372)	.95
Ease of use	0.64 (0.04; 0.56 to 0.71)	15.87 (372)	.001
Anxiety	−0.10 (0.05; −0.19 to −0.01)	−2.09 (372)	.03
Ease of use × anxiety	−0.07 (0.03; −0.14 to −0.004)	−2.08 (372)	.03
Conditional effects of ease of use on intention at different anxiety levels			
Low	0.71 (0.05; 0.62 to 0.81)	14.51 (372)	<.001
Mean	0.62 (0.04; 0.54 to 0.71)	15.00 (372)	<.001
High	0.56 (0.06; 0.45 to 0.68)	9.55 (372)	<.001
Conditional indirect effects at different anxiety levels			
Low	−0.20 (0.05; −0.29 to −0.11)	—^a^	—
Mean	−0.17 (0.04; −0.26 to −0.09)	—	—
High	−0.15 (0.04; −0.24 to −0.08)	—	—

a Not applicable.

Emotional reactivity was negatively associated with ease of use (*B*=−0.27, SE=0.05, *t*_375_=−5.38; *P*<.001), which in turn was positively associated with intention to use (*B*=0.64, SE=0.04, *t*_372_=15.87; *P*<.001). The significance of the mediation effect was estimated using 95% CIs calculated based on 5000 bootstrap samples. Since zero was not included in the CIs at all levels of anxiety, the indirect relationships were significant across all anxiety levels.

The interaction between ease of use and anxiety was significant (*B*=−0.07, SE=0.03, *t*_372_=−2.08; *P*=.04). The conditional effects show that ease of use remains positively associated with intention to use across all anxiety levels: low anxiety (*B*=0.71, SE=0.05, *t*_372_=14.51; *P*<.001), mean anxiety (*B*=0.62, SE=0.04, *t*_372_=15.00; *P*<.001), and high anxiety (*B*=0.56, SE=0.06, *t*_372_=9.55; *P*<.001).

The conditional indirect relationships show that the negative effect of emotional reactivity on intention to use through reduced ease of use weakens as anxiety increases: low anxiety (indirect effect=−0.20, 95% CI −0.29 to −0.11), mean anxiety (indirect effect=−0.17, 95% CI −0.26 to −0.09), and high anxiety (indirect effect=−0.15, 95% CI −0.24 to −.08). All indirect effects remained statistically significant across anxiety levels, indicating that while anxiety moderates the strength of the indirect effect, it cannot eliminate it entirely.

The index of moderated mediation for the indirect relationship between short-term focus and intention to use through ease of use, with anxiety as the moderator, was statistically significant (index=0.021, 95% CI 0.004-0.044), supporting the presence of a moderated indirect effect. Table S2 in Multimedia Appendix 1 presents a detailed description of all analysis steps.

Short-term focus was negatively associated with ease of use (*B*=−0.30, SE=0.05, *t*_375_=−5.86; *P*<.001), which in turn was positively associated with intention to use (*B*=0.64, SE=0.04, *t*_372_=15.80; *P*<.001). The significance of the mediation effect was estimated using 95% CIs calculated based on 5000 bootstrap samples. Since zero was not included in the CIs at all levels of anxiety, the indirect relationships were significant across all anxiety levels.

The interaction between ease of use and anxiety was significant (*B*=−0.07, SE=0.03, *t*_372_=−2.07; *P*=.04). The conditional effects show that ease of use remains positively associated with intention to use across all anxiety levels: low anxiety (*B*=0.71, SE=0.05, *t*_372_=14.24; *P*<.001), mean anxiety (*B*=0.62, SE=0.04, *t*_372_=14.96; *P*<.001), and high anxiety (*B*=0.56, SE=0.06, *t*_372_=9.56; *P*<.001). The conditional indirect relationships show that the negative effect of short-term focus on intention to use through reduced ease of use weakens as anxiety increases: low anxiety (indirect effect=−0.21, 95% CI −0.31 to −0.12), mean anxiety (indirect effect=−0.18, 95% CI −0.27 to −0.10), and high anxiety (indirect effect=−0.17, 95% CI −0.25 to −0.09). All indirect effects remained statistically significant across anxiety levels, indicating that while anxiety moderates the strength of the indirect effect, it cannot eliminate it entirely.

The index of moderated mediation for the indirect relationship between cognitive rigidity and intention to use through ease of use, with anxiety as the moderator, was statistically significant (index=−0.020, 95% CI −0.043 to −0.004), supporting the presence of a moderated indirect effect. Table 5 presents a detailed description of all analysis steps.

**Table 5.** Moderated mediation model predicting intention to use through ease of use, with anxiety as moderator.

Model component	*B* (SE; 95% CI)	*t* test (*df*)	*P* value
Predicting the mediator: ease of use			
Cognitive rigidity	0.27 (0.05; 0.17 to 0.37)	5.27 (375)	<.001
Predicting the dependent variable: intention to use			
Cognitive rigidity	−0.07 (0.04; −0.15 to 0.01)	−1.71 (372)	.08
Ease of use	0.66 (0.04; 0.58 to 0.74)	15.82 (372)	<.001
Anxiety	−0.08 (0.04; −0.16 to −0.0004)	−1.98 (372)	.04
Ease of use × anxiety	−0.08 (0.03; −0.14 to −0.01)	−2.24 (372)	.02
Conditional effects of ease of use on intention at different anxiety levels			
Low	0.74 (0.05; 0.64 to 0.84)	14.48 (372)	<.001
Mean	0.64 (0.04; 0.56 to 0.73)	15.02 (372)	<.001
High	0.58 (0.06; 0.46 to 0.69)	9.75 (372)	<.001
Conditional indirect effects at different anxiety levels			
Low	0.20 (0.04; 0.12 to 0.28)	—^a^	—
Mean	0.17 (0.04; 0.10 to 0.25)	—	—
High	0.16 (0.04; 0.09 to 0.23)	—	—

a Not applicable.

Cognitive rigidity was positively associated with ease of use (*B*=0.27, SE=0.05, *t*_375_=5.27; *P*<.001), which in turn was positively associated with intention to use (*B*=0.66, SE=0.04, *t*_372_=15.82; *P*<.001). The significance of the mediation effect was estimated using 95% CIs calculated based on 5000 bootstrap samples. Since zero was not included in the CIs at all levels of anxiety, the indirect relationships were significant across all anxiety levels.

The interaction between ease of use and anxiety was significant (*B*=–0.08, SE=0.03, *t*_372_=–2.24; *P*=.02). The conditional effects show that ease of use remains positively associated with intention to use across all anxiety levels: low anxiety (*B*=0.74, SE=0.05, *t*_372_=14.48; *P*<.001), mean anxiety (*B*=0.64, SE=0.04, *t*_372_=15.02; *P*<.001), and high anxiety (*B*=0.58, SE=0.06, *t*_372_=9.75; *P*<.001). The conditional indirect relationships show that the positive effect of cognitive rigidity on intention to use through increased ease of use weakens as anxiety increases: low anxiety (indirect effect=0.20, 95% CI 0.12-0.28), mean anxiety (indirect effect=0.17, 95% CI 0.10-0.25), and high anxiety (indirect effect=0.16, 95% CI 0.09-0.23). All indirect effects remained statistically significant across anxiety levels, indicating that while anxiety moderates the strength of the indirect effect, it cannot eliminate it entirely.

## Discussion

### Principal Findings

The results of the moderated-mediation analyses fit the proposed model: caregivers with stronger dispositional RTC reported weaker intention to use telemedicine, and this link was explained mainly by their lower ease of use. The indirect link was weaker when telemedicine-related anxiety was higher, but it stayed statistically significant across the whole range of anxiety observed. Since the data were collected at 1 point in time and rely on self-report, these findings describe associations and not proven cause and effect. The overall indirect effect of RTC remained negative and statistically significant across all anxiety levels. This profile dovetails with provider-side applications of the TAM, in which PEU has consistently been found to mediate associations between inhibiting traits and usage intentions among nurses, physicians, and allied professionals [40,41].

The buffering role of anxiety accords with hospital studies in which technology-related anxieties were associated with a weaker link between usability perceptions and usage intentions. Kummer et al [33], for example, found that anxieties about a sensor-based medication system suppressed the PEU-intention link among ward staff; our data show a comparable attenuation and are consistent with PEU operating as a mediator even among highly apprehensive users. These findings add to recent TAM extensions by showing that RTC was associated with intention mainly through PEU and that the pattern differed across RTC dimensions. Routine seeking, emotional reactivity, and short-term focus followed the expected negative pathway, whereas cognitive rigidity did not. This suggests that resistance should not be treated as a single, uniform barrier in technology-acceptance models. Telemedicine-related anxiety changed the strength of the PEU-intention association but did not remove it, supporting its role as a boundary condition of the indirect pathway.

The dimension-specific results provide further detail. Routine seeking produced the largest conditional indirect effect, dropping from –0.22 at low anxiety to –0.18 at high anxiety. It is noteworthy that while the simple interaction effect for routine seeking was marginally nonsignificant, the index of moderated mediation remained significant, consistent with Hayes’ [39] argument that the bootstrap-based index provides a more sensitive test for detecting moderation in indirect pathways. Prior evidence from electronic-record roll-outs shows that clinicians who prize habitual workflows are especially sensitive to any additional cognitive effort a new system entails, consistent with our finding that PEU accounted for much of the association in this group [40].

Emotional reactivity and short-term focus followed the same gradient, falling from –0.20 to –0.15 and from –0.21 to –0.17, respectively. Both dimensions capture immediate affective or workload costs, which inflate perceived difficulty during the early learning curve and thus translate into lower intention to use. Reports of caregivers abandoning teleconsultation platforms after initial frustration with interface complexity mirror this mechanism [42].

Unlike the other RTC dimensions, cognitive rigidity was positively associated with PEU (*B*=0.27), and the corresponding indirect association with intention to use decreased from 0.20 at low anxiety to 0.16 at high anxiety. This direction was contrary to H1 and to the usual interpretation of cognitive rigidity as a barrier to change [13]. The result should therefore be treated with caution. One possible explanation is that some telemedicine procedures follow fixed and rule-based steps that may appear straightforward to professionals who prefer structured working methods. However, the present study did not assess task-technology fit or preferences for structured workflows, and the study by Cady and Finkelstein [34] did not examine cognitive rigidity directly. The finding may also reflect characteristics of the present sample or the modified cognitive rigidity subscale. It does not show that cognitive rigidity facilitates telemedicine use and should be replicated in other samples [34]. Across the RTC dimensions, the PEU × telemedicine-related anxiety interaction was statistically significant in the emotional reactivity, short-term focus, and cognitive rigidity models, but not in the routine-seeking model. The conditional indirect associations nevertheless remained statistically significant at all examined anxiety levels. This suggests that PEU may remain relevant even among professionals reporting higher telemedicine-related anxiety. Routine seeking, emotional reactivity, and short-term focus were associated with negative indirect pathways of different magnitudes. These findings may inform future intervention research, but the present study did not evaluate any tailored strategy or its effectiveness. Because the cognitive rigidity finding was unexpected, no specific practical recommendation should be based on it at this stage.

### Limitations and Future Research

The findings need to be interpreted in light of several limitations. Because the data were collected at 1 time point, the temporal order of RTC, PEU, telemedicine-related anxiety, and intention to use cannot be established. The indirect associations identified in the PROCESS analyses therefore do not demonstrate causal pathways. Longitudinal research conducted before and after telemedicine implementation would help clarify how these relationships develop over time.

All variables were measured by self-report in the same questionnaire. The results may therefore have been affected by common method bias, shared measurement variance, or socially desirable responding. The study also examined intention to use telemedicine rather than actual use. Future research could combine questionnaires with system logs or administrative records.

Participants were recruited from 4 governmental GMCs within 1 national health system, and the final sample represented 31.8% (n=377) of the eligible workforce. Although staff were approached across departments and work shifts, participation was voluntary, and those who participated may have differed from those who did not. The findings may therefore not apply to other health care settings, countries, professional groups, or telemedicine systems. In addition, telemedicine use in the participating centers was limited, optional, and mainly related to remote patient monitoring. The questionnaire did not distinguish among specific telemedicine modalities or interaction structures, so modality-specific analyses could not be conducted. The findings should therefore be interpreted primarily in relation to this context. Future studies should examine whether the observed associations differ across audio-only, video-based, remote-monitoring, and hybrid models and between dyadic and triadic interactions.

The positive association found for cognitive rigidity also requires caution. One item was removed from this subscale, and the direction of the result was contrary to the original hypothesis. This finding should be examined again in other samples and with additional assessment of cognitive rigidity.

### Conclusions

This study indicates that clinicians’ RTC was associated with weaker intention to use telemedicine, an association statistically accounted for by lower ease of use, and that this indirect association was smaller at higher levels of telemedicine-related anxiety while remaining significant throughout the observed range (−0.17 at low anxiety vs −0.14 at high). Routine seeking, emotional reactivity, and short-term focus were each associated with the negative pathway. Cognitive rigidity showed an unexpected positive association with PEU and a positive indirect association with intention to use. This finding should be treated as preliminary and replicated before practical implications are drawn from it. The findings extend dual-factor approaches to technology acceptance by showing that RTC dimensions did not follow a uniform pattern and that telemedicine-related anxiety conditioned the indirect association between RTC and intention to use through PEU. The findings suggest that RTC and PEU may warrant consideration when developing strategies to support telemedicine use intentions in geriatric practice.

## Supplementary material

10.2196/95371 Multimedia Appendix 1 Supplementary moderated mediation analyses for the routine seeking and short-term focus dimensions of resistance to change.

10.2196/95371 Checklist 1 STROBE checklist.

## Abbreviations

GMC: geriatric medical center
PEU: ease of use
RTC: resistance to change
STROBE: Strengthening the Reporting of Observational Studies in Epidemiology
TAM: Technology Acceptance Model
VIF: variance inflation factor

## References

[1] Singh V, Dev V (2021). Telemedicine adoption in India: identifying factors affecting intention to use. Int J Healthc Inf Syst Inform.

[2] Stoumpos AI, Kitsios F, Talias MA (2023). Digital transformation in healthcare: technology acceptance and its applications. Int J Environ Res Public Health.

[3] Bocean CG, Vărzaru AA (2025). Health status in the era of digital transformation and sustainable economic development. BMC Health Serv Res.

[4] Türkyılmaz S (2023). Investigation of telemedicine services, an innovative and technology-based healthcare application, by using the extended Technology Acceptance Model (TAM2): an example from Turkey. Eur J Appl Sci.

[5] Alotaibi YK, Federico F (2017). The impact of health information technology on patient safety. Saudi Med J.

[6] (2010). Telemedicine: opportunities and developments in Member States: report on the Second Global Survey on eHealth 2009. https://iris.who.int/server/api/core/bitstreams/1d9e2557-20e2-4ab4-bdd7-648f962f866d/content.

[7] Bashshur RL, Howell JD, Krupinski EA, Harms KM, Bashshur N, Doarn CR (2016). The empirical foundations of telemedicine interventions in primary care. Telemed J E Health.

[8] Sedláček D, Lochmannová A, Šín R, Křivková J, Kovalčinová K, Marek P (2025). Frontline ethical burdens: a mixed‑methods investigation of moral distress in emergency medical services. Bratisl Med J.

[9] Sharon C, Hochwald IH, Green G, Lochmannová A (2026). Technology-enriched high-fidelity simulation in nursing education: learning effectiveness and mediating model. Med Sci Educ.

[10] Davis FD (1989). Perceived usefulness, perceived ease of use, and user acceptance of information technology. MIS Q.

[11] Schürmann F, Westmattelmann D, Schewe G (2025). Factors influencing telemedicine adoption among health care professionals: qualitative interview study. JMIR Form Res.

[12] Laumer S, Maier C, Eckhardt A (2010). Proceedings of the 18th European Conference on Information Systems (ECIS).

[13] Oreg S (2006). Personality, context, and resistance to organizational change. Eur J Work Organ Psychol.

[14] Tsai TH, Lin WY, Chang YS, Chang PC, Lee MY (2020). Technology anxiety and resistance to change behavioral study of a wearable cardiac warming system using an extended TAM for older adults. PLoS One.

[15] Lochmannová A (2025). Exploring the role of virtual reality in preparing emergency responders for mass casualty incidents. Isr J Health Policy Res.

[16] Bednar M, Dufek P, Lochmannova A, Simon M, Bures M (2023). Use of virtual reality for education and training of emergency rescue system for crisis situations. Proc Int Conf Educ Technol Comput.

[17] Kautish P, Siddiqui M, Siddiqui A, Sharma V, Alshibani SM (2023). Technology-enabled cure and care: an application of innovation resistance theory to telemedicine apps in an emerging market context. Technol Forecast Soc Change.

[18] Oreg S (2003). Resistance to change: developing an individual differences measure. J Appl Psychol.

[19] Oreg S, Bayazit M, Vakola M (2008). Dispositional resistance to change: measurement equivalence and the link to personal values across 17 nations. J Appl Psychol.

[20] Verpaalen IAM, Holland RW, Ritter S (2022). Resistance to contact tracing applications: the implementation process in a social context. Comput Human Behav.

[21] Kotter JP (1996). Leading Change.

[22] Özdemir-Güngör D, Camgöz-Akdağ H (2018). Examining the effects of technology anxiety and resistance to change on the acceptance of breast tumor registry system: evidence from Turkey. Technol Soc.

[23] Fahmi I (2022). The antecedents of intention to use telemedicine. J Consum Sci.

[24] Tsai JM, Cheng MJ, Tsai HH, Hung SW, Chen YL (2019). Acceptance and resistance of telehealth: the perspective of dual-factor concepts in technology adoption. Int J Inf Manage.

[25] Safi S, Thiessen T, Schmailzl KJ (2018). Acceptance and resistance of new digital technologies in medicine: qualitative study. JMIR Res Protoc.

[26] Amarantou V, Kazakopoulou S, Chatzoudes D, Chatzoglou P (2018). Resistance to change: an empirical investigation of its antecedents. J Organ Change Manag.

[27] Venkatesh V, Morris MG, Davis GB, Davis FD (2003). User acceptance of information technology: toward a unified view. MIS Q.

[28] Yeow YKA, Lim WK (2017). Resisting technology-enabled change within healthcare organization. Acad Manag Proc.

[29] Vadakkemulanjanal Joseph G, Anit Thomas M (2022). The mediation effect of technology anxiety and barriers on technology exposure to teachers’ technology adoption. Proc 3rd Int Conf Intell Comput Instrum Control Technol.

[30] Nestik T, Zhuravlev A, Patrakov E (2019). Technophobia as a cultural and psychological phenomenon. Interação Rev Ensino Pesqui Ext.

[31] Haig EL, Woodcock KA (2017). Rigidity in routines and the development of resistance to change in individuals with Prader-Willi syndrome. J Intellect Disabil Res.

[32] Samhan B (2025). Proc Hawaii Int Conf Syst Sci.

[33] Kummer TF, Recker J, Bick M (2017). Technology-induced anxiety: manifestations, cultural influences, and its effect on the adoption of sensor-based technology in German and Australian hospitals. Inf Manag.

[34] Cady RG, Finkelstein SM (2014). Task-technology fit of video telehealth for nurses in an outpatient clinic setting. Telemed J E Health.

[35] Green G, Lochmannová A, Porat-Packer T (2026). The pathway from loneliness to chronic diseases: examining the mediating roles of depression and self-perceived health, and the moderating effect of age among older adults in Israel. J Appl Gerontol.

[36] Green G (2025). Electronic health literacy among older adults: development and psychometric validation of the Hebrew version of the electronic health literacy questionnaire. Int J Med Inform.

[37] Zangirolami-Raimundo J, Echeimberg JDO, Leone C (2018). Research methodology topics: cross-sectional studies. J Hum Growth Dev.

[38] von Elm E, Altman DG, Egger M (2008). The Strengthening the Reporting of Observational Studies in Epidemiology (STROBE) statement: guidelines for reporting observational studies. J Clin Epidemiol.

[39] Hayes AF (2022). Introduction to Mediation, Moderation, and Conditional Process Analysis: A Regression-Based Approach.

[40] Cho Y, Kim M, Choi M (2021). Factors associated with nurses’ user resistance to change of electronic health record systems. BMC Med Inform Decis Mak.

[41] Kung LH, Yan YH, Kung CM (2024). Exploring telemedicine usage intention using technology acceptance model and social capital theory. Healthcare (Basel).

[42] Diel S, Doctor E, Reith R, Buck C, Eymann T (2023). Examining supporting and constraining factors of physicians’ acceptance of telemedical online consultations: a survey study. BMC Health Serv Res.

