# Supplementary material for: Telemedicine Adoption Among Health Care Professionals in Israeli Geriatric Medical Centers: Moderated Mediation Cross-Sectional Questionnaire Study
Source: JMIR Hum Factors. 2026 Oct 5;13:e95371. doi: 10.2196/95371 (PMC13637990; doi:10.2196/95371)
Supplement: Supplementary file 1 [file humanfactors-v13-e95371-s001.docx]

**Table S1.** Moderated Mediation Model Predicting Intention to use Through Ease of Use, with Anxiety as Moderator

|  | **B** | **SE** | **t** | **P value** | **95% CI** |
| --- | --- | --- | --- | --- | --- |
| **Predicting the mediator: Ease of Use** |  |  |  |  |  |
| Routine Seeking | -.32 | .05 | -6.41 | *** | [-.42, -.22] |
|  |  |  |  |  |  |
| **Predicting the Dependent Variable: Intention to use** |  |  |  |  |  |
| Routine Seeking | -.08 | .05 | -1.82 | n.s | [-.17, .01] |
| Ease of Use | .62 | .04 | 15.50 | *** | [.54, .70] |
| Anxiety | -.06 | .04 | -1.32 | n.s | [-.14, .03] |
| Ease of Use × Anxiety | -.06 | .03 | -1.83 | n.s | [-.13, .00] |
|  |  |  |  |  |  |
| **Conditional Effects of Ease of Use on Intention at Different Anxiety Levels** |  |  |  |  |  |
| Low | .69 | .05 | 13.84 | *** | [.59, .79] |
| Mean | .61 | .04 | 14.71 | *** | [.53, .69] |
| High | .56 | .06 | 9.53 | *** | [.44, .67] |
|  |  |  |  |  |  |
| **Conditional Indirect Effects at Different Anxiety Levels** |  |  |  |  |  |
| Low | -.22 | .04 | -- | -- | [-.30, -.15] |
| Mean | -.20 | .04 | -- | -- | [-.27, -.13] |
| High | -.18 | .04 | -- | -- | [-.26, -.11] |

Note: *P < .05., **P < .01, ***P<0.001. n.s= not significant

**Table S2.** Moderated Mediation Model Predicting Intention to use Through Ease of Use, with Anxiety as Moderator

|  | **B** | **SE** | **t** | **P value** | **95% CI** |
| --- | --- | --- | --- | --- | --- |
| **Predicting the mediator: Ease of Use** |  |  |  |  |  |
| Short-term Focus | -.30 | .05 | -5.86 | *** | [-.40, -.20] |
|  |  |  |  |  |  |
| **Predicting the Dependent Variable: Intention to use** |  |  |  |  |  |
| Short-term Focus | .01 | .04 | .14 | n.s | [-.08, .09] |
| Ease of Use | .64 | .04 | 15.80 | *** | [.56, .72] |
| Anxiety | -.10 | .04 | -2.28 | .* | [-.18, -.01] |
| Ease of Use × Anxiety | -.07 | .03 | -2.07 | * | [-.14, -.004] |
|  |  |  |  |  |  |
| **Conditional Effects of Ease of Use on Intention at Different Anxiety Levels** |  |  |  |  |  |
| Low | .71 | .05 | 14.24 | *** | [.61, .81] |
| Mean | .62 | .04 | 14.96 | *** | [.54, .71] |
| High | .56 | .06 | 9.56 | *** | [.45, .68] |
|  |  |  |  |  |  |
| **Conditional Indirect Effects at Different Anxiety Levels** |  |  |  |  |  |
| Low | -.21 | .05 | -- | -- | [-.31, -.12] |
| Mean | -.18 | .04 | -- | -- | [-.27, -.10] |
| High | -.17 | .04 | -- | -- | [-.25, -.09] |

Note: *P < .05., **P < .01, ***P<0.001. n.s= not significant
