# Supplementary material for: Telemedicine Adoption Among Health Care Professionals in Israeli Geriatric Medical Centers: Moderated Mediation Cross-Sectional Questionnaire Study
Source: JMIR Hum Factors. 2026 Oct 5;13:e95371. doi: 10.2196/95371 (PMC13637990; doi:10.2196/95371)
Supplement: Supplementary file 2 [file humanfactors-v13-e95371-s002.docx]

**STROBE Statement—Checklist of Items That Should Be Included in Reports of Cross-Sectional Studies**

| **Section** | **Item** | **STROBE recommendation** | **Reported on page(s) / section** |
| --- | --- | --- | --- |
| Title and abstract | 1(a) | Indicate the study's design with a commonly used term in the title or the abstract. | p. 1 (Title and Abstract) |
| Title and abstract | 1(b) | Provide in the abstract an informative and balanced summary of what was done and what was found. | p. 1 (Abstract) |
| Introduction | 2 | Explain the scientific background and rationale for the investigation being reported. | pp. 2-4 (Introduction) |
| Introduction | 3 | State specific objectives, including any prespecified hypotheses. | pp. 4-5 (Research question and hypotheses) |
| Methods | 4 | Present key elements of the study design early in the paper. | p. 5 (Methods paragraph) |
| Methods | 5 | Describe the setting, locations, and relevant dates, including periods of recruitment, exposure, follow-up, and data collection. | pp. 5-7 (Participants and Procedure) |
| Methods | 6(a) | Give the eligibility criteria, and the sources and methods of selection of participants. | pp. 5-6 (Participants) |
| Methods | 7 | Clearly define all outcomes, exposures, predictors, potential confounders, and effect modifiers. Give diagnostic criteria, if applicable. | pp. 4-5 and 7-9 (Hypotheses and Instruments); diagnostic criteria not applicable |
| Methods | 8 | For each variable of interest, give sources of data and details of methods of assessment (measurement). Describe comparability of assessment methods if there is more than one group. | pp. 7-9 (Instruments) |
| Methods | 9 | Describe any efforts to address potential sources of bias. | pp. 6-9 and 22 (recruitment across departments and shifts, anonymous independent administration, translation and pilot testing, and limitations) |
| Methods | 10 | Explain how the study size was arrived at. | pp. 5-6 (the entire eligible workforce was approached; 377 of 1,184 professionals participated) |
| Methods | 11 | Explain how quantitative variables were handled in the analyses. If applicable, describe which groupings were chosen and why. | pp. 7-9 and 12-19 (scale scoring, standardization, and anxiety levels at the mean and 1 SD below/above the mean) |
| Methods | 12(a) | Describe all statistical methods, including those used to control for confounding. | pp. 9-10 (Statistical Analysis); pp. 12-19 (bootstrap confidence intervals and model estimates) |
| Methods | 12(b) | Describe any methods used to examine subgroups and interactions. | pp. 9-10 (separate analyses by resistance-to-change dimension and the ease-of-use by anxiety interaction) |
| Methods | 12(c) | Explain how missing data were addressed. | p. 9 (no item-level or scale-level missing data were identified) |
| Methods | 12(d) | If applicable, describe analytical methods taking account of the sampling strategy. | Not applicable: the study used a census-style invitation without complex sampling or weighting. |
| Methods | 12(e) | Describe any sensitivity analyses. | Not applicable: no sensitivity analyses were planned or performed. |
| Results | 13(a) | Report numbers of individuals at each stage of the study, for example numbers potentially eligible, examined for eligibility, confirmed eligible, included in the study, and analysed. | pp. 5-6 and 9-10 (1,184 in the target population; 377 completed questionnaires; all 377 analysed) |
| Results | 13(b) | Give reasons for non-participation at each stage. | p. 6 (eligibility and exclusion criteria); individual reasons for non-participation were not recorded. |
| Results | 13(c) | Consider use of a flow diagram. | Not applicable: recruitment involved one cross-sectional invitation stage and no flow diagram was used. |
| Results | 14(a) | Give characteristics of study participants and information on exposures and potential confounders. | pp. 10-12 (Table 1 and Table 2) |
| Results | 14(b) | Indicate the number of participants with missing data for each variable of interest. | p. 9 (no missing data; all 377 cases contributed to every analysis) |
| Results | 15 | Report numbers of outcome events or summary measures. | pp. 10-19 (Tables 1-7 and Figures 1-3) |
| Results | 16(a) | Give unadjusted estimates and, if applicable, confounder-adjusted estimates and their precision, such as 95% confidence intervals. Make clear which confounders were adjusted for and why they were included. | pp. 12-19 (B, SE, t, P values, and 95% confidence intervals); the prespecified models did not include covariates. |
| Results | 16(b) | Report category boundaries when continuous variables were categorized. | pp. 14-19 (conditional effects at the mean and 1 SD below/above the mean of standardized anxiety) |
| Results | 16(c) | If relevant, consider translating estimates of relative risk into absolute risk for a meaningful time period. | Not applicable: no relative-risk estimates or time-to-event outcomes were analysed. |
| Results | 17 | Report other analyses done, for example analyses of subgroups and interactions, and sensitivity analyses. | pp. 15-19 (dimension-specific moderated mediation and interaction analyses); no sensitivity analyses were performed. |
| Discussion | 18 | Summarize key results with reference to study objectives. | pp. 20-23 (Discussion and Conclusions) |
| Discussion | 19 | Discuss limitations of the study, taking into account sources of potential bias or imprecision. Discuss both direction and magnitude of any potential bias. | p. 22 (Limitations and Future Research) |
| Discussion | 20 | Give a cautious overall interpretation of results considering objectives, limitations, multiplicity of analyses, results from similar studies, and other relevant evidence. | pp. 20-23 (Discussion and Conclusions) |
| Discussion | 21 | Discuss the generalizability (external validity) of the study results. | p. 22 (Limitations and Future Research) |
| Other information | 22 | Give the source of funding and the role of the funders for the present study and, if applicable, for the original study on which the present article is based. | p. 23 (Funding); the funding source is reported, but the funder's role is not specified. |
